# Expression of human Krüppel‐like factor 3 in peripheral blood as a promising biomarker for acute leukemia

**DOI:** 10.1002/cam4.2911

**Published:** 2020-02-26

**Authors:** Miao Yan, Huihui Liu, Junhui Xu, Xinan Cen, Qian Wang, Weilin Xu, Wensheng Wang, Zhixiang Qiu, Jinping Ou, Yujun Dong, Ping Zhu, Hanyun Ren, Fuchu He, Mangju Wang

**Affiliations:** ^1^ Department of Hematology Peking University First Hospital Beijing China; ^2^ State Key Laboratory of Proteomics Beijing Proteome Research Center Institute of Radiation Medicine Academy of Military Medical Sciences Beijing China

**Keywords:** acute leukemia, biomarker, diagnosis, human krüppel‐like factor 3, minimal residual disease

## Abstract

**Background:**

Universal gene targets are in persistent demand by real‐time quantitative polymerase chain reaction (RT‐qPCR)‐based methods in acute leukemia (AL) diagnosis and monitoring. Human Krüppel‐like factor 3 (hKLF3), a newly cloned human transcription factor, has proved to be a regulator of hematopoiesis.

**Methods:**

Sanger sequencing was performed in bone marrow (BM) samples from 17 AL patients for mutations in hKLF3 coding exons. hKLF3 expression in peripheral blood (PB) and BM samples from 45 AL patients was dynamically detected by RT‐qPCR. PB samples from 31 healthy donors were tested as normal controls.

**Results:**

No mutation was sequenced in hKLF3 coding exons. hKLF3 expression in PB of AL was significantly lower than that in healthy donors [0.30 (0.02‐1.07) vs 1.18 (0.62‐3.37), *P* < .0001]. Primary acute myeloid leukemia (AML) exhibited the least expression values compared with secondary AML and acute lymphoblastic leukemia. Receiver operating characteristic (ROC) analyses suggested that hKLF3 expression in PB was a good marker for AML diagnosis with an AUC of 0.99 (95% CI 0.98‐1.00) and an optimum cutoff value of 0.67 (sensitivity 93.94% and specificity 93.55%). hKLF3 expression was upregulated significantly when AML patients acquired morphological complete remission (CR), and the level of hKLF3 seemed to be higher in patients with deeper CR than in patients with minimal residual disease (MRD). Paired PB and BM samples showed highly consistent alteration in hKLF3 expression (*r* = .6533, *P* = .001). Besides, a significantly converse correlation between decreased hKLF3 expression in PB and markers for leukemic load was observed.

**Conclusions:**

hKLF3 expression in PB may act as a potential marker for AL diagnosis and monitoring.

## Graphical abstract

In the present study, we investigated the expression of hKLF3 in acute leukemia patients by both Sanger sequencing and RT‐qPCR. ROC analyses indicated excellent efficacy of hKLF3 expression in PB samples for the diagnosis of AML. In addition, a significantly converse correlation between inhibition of hKLF3 expression in peripheral blood and increased leukemic burden was observed.

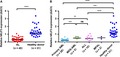

## 1 INTRODUCTION

The diagnosis and risk evaluation of acute leukemia (AL) is currently based on morphology, immunology, cytogenetics, and molecular biology. Detection of gene abnormality through real‐time quantitative polymerase chain reaction (RT‐qPCR) is a significant method in the diagnosis of AL, evaluation of chemotherapy response, as well as monitoring of minimal residual disease (MRD). The PCR‐based method is highly sensitive, rapid, and well standardized, but it is dependent on specific gene targets. However, currently, most gene targets are not available on as more as 50% of patients,^1^ so it is in persistent demand to identify molecular targets applicable for the majority of patients. Researchers have been investigating gene targets whose expression is extensive but significantly changed in AL blasts relative to normal peripheral blood (PB) and bone marrow (BM).

The human Krüppel‐like factor 3 (hKLF3), a newly cloned human transcriptional factor from the cDNA library of 22‐week fetal liver in our previous work,^2^ belongs to the Krüppel‐like transcription factor family.^3^ hKLF3 contains two transcripts. The larger one exists widely in various tissues, while the smaller one mainly expresses in PB leukocytes, liver, and BM.^2^ Its expression level in fetal livers decreased as fetal livers developed into adult livers, during which the hematopoietic function gradually disappeared, and the expression level of this factor kept increasing during the process of erythrocytes and granulocytes maturation,^2^ which indicated the role of hKLF3 in hematopoiesis regulation. Additionally, our preliminary research has confirmed that hKLF3 inhibited proliferation of K562 cells.^4^ Taken together, hKLF3 was assumed to be an essential transcription factor involved in the proliferation and differentiation regulation of hematopoietic cells. As AL is characterized by limitless proliferation and disfunction of differentiation of leukemic blasts, we assume that hKLF3 expression in leukemic cells is different from normal hematopoietic samples, and the extensive expression of hKLF3 in hematopoietic cells would enable it to function as a promising universal target for PCR‐based methods in the diagnosis and monitoring of hematological malignant diseases.

Currently, BM specimens are the main detection objects for the molecular diagnosis and monitoring of leukemia. With easy availability and minimal trauma, detections for molecular markers in PB samples are thought to be good alternatives. In our previous work, we have been exploring the value of PB samples in molecular diagnosis of leukemia. The aim of this study was to evaluate the possibility of exploiting hKLF3’s expression in PB samples for a new molecular marker in the diagnosis and monitoring of AL. Firstly, we sequenced coding exons of hKLF3 in BM samples from AL patients by Sanger sequencing for gene mutations. And in the meantime, we detected the hKLF3 expression levels in AL patients, respectively, at the time of diagnosis and after morphological complete remission (CR), and determined the optimum cutoff value of hKLF3 expression in PB for the criteria to distinguish AL from healthy controls. Then, we compared the consistency of the factor's change in paired PB and BM samples. In the end, the correlation between the expression values of hKLF3 and the tumor burden was analyzed.

## 2 MATERIALS AND METHODS

### 2.1 Study population and samples

All of the 45 newly diagnosed AL patients and 31 healthy stem cell donors in the Hematology Department of Peking University First Hospital from 1 October 2018 to 31 August 2019 were recruited in the study. The subtypes of AL according to tumor origin were as follows: 33 cases of acute myeloid leukemia (AML), 10 cases of acute lymphoblastic leukemia (ALL), and two cases of mixed phenotypic acute leukemia (MPAL). The diagnosis of AL and evaluation of morphological CR were based on the 2016 WHO criterion. Table 1 summarized the demographic characteristics and clinical features of the recruited population. The percentage of blast cells in PB was determined by microscopic observation, and the percentage of blast cells in BM and MRD after chemotherapy was determined by flow cytometry (FCM). Forty‐one of the 45 AL patients underwent standard chemotherapy, of which 32 achieved morphological CR by the end of our follow‐up in 31 August 2019. PB samples at diagnosis from all the AL patients and PB samples at morphological CR from 20 patients out of the total were collected into EDTA tubes. BM samples at the time of diagnosis from 22 patients were also collected. All the healthy donors had no malignant disease history, and PB samples were taken from them before hematopoietic stem cell mobilization. The study was conducted in accordance with the Declaration of Helsinki and reviewed by the institutional ethics review committee of Peking University First Hospital. All participants provided written informed consent.

**Table 1:** Characteristics of the studied population

	donors n = 31	AL		AML		ALL	
		n = 45	*P*	n = 33	*P*	n = 10	*P*
Gender (male/female)	20/11	30/15	.846	25/8	.365	5/5	.413
Age (y)	37 ± 9	44 (16‐72)	.105	50 (19‐72)	.001	28 (16‐63)	.046
WBC count (×10^9^/L)	5.46 ± 1.13	5.00 (0.80‐211.00)	.915	7.05 (0.80‐211.00)	.386	2.95 (1.00‐110.40)	.259
HGB (g/L)	137 ± 15	80 (43‐154)	.000	78 (46‐142)	.000	88 (43‐154)	.000
PLT count (×10^9^/L)	226 ± 57	43 (4‐1410)	.000	39 (4‐1410)	.000	65 (19‐200)	.000
LDH (IU/L)	159 ± 26	310 (119‐1452)	.000	322 (143‐1452)	.000	364 (119‐1331)	.006
PB blasts (%)	NA	66.50 (2.00‐98.00)	NA	66.50 (19.00‐98.00)	NA	55.00 (2.00‐91.00)	NA
BM blasts by FCM (%)	NA	66.42 (5.23‐96.39)	NA	53.88 (5.23‐96.39)	NA	84.03 (11.69‐92.67)	NA

Note

Data were presented as median (range) or mean ± SD.

Abbreviations: AL, acute leukemia; ALL, acute lymphoblastic leukemia; AML, acute myeloid leukemia; BM, bone marrow; FCM, flow cytometry; HGB, hemoglobin; hKLF3, human Krüppel‐like factor 3; LDH, lactate dehydrogenase; NA, not applicable; PB, peripheral blood; PLT, platelet; SD, standard deviation; WBC, white blood cell.

### 2.2 Sanger sequencing of hKLF3 coding exons

DNA was extracted from BM samples using a TIANamp Genomic DNA Kit in accordance with manufacturer's protocol (TIANGEN BIOTECH). NanoDrop 2000 (Thermo Scientific) was used to quantify and assess the purity and concentration of isolated DNA. Coding sequence of hKLF3, which spans exon 2‐6, was amplified in a 30‐µL reaction system (TIANGEN BIOTECH). The PCR products were electrophoresed and purified according to manufacturer's protocol (Millipore). Finally, the DNA sequencing reaction was performed with the BGI 2× Super PCR Mix with dye (BGI) with an ABI 3730XL sequence detection system (Applied Biosystems). The primer sequences for amplification and sequencing reaction are shown in the Supporting Information.

### 2.3 hKLF3 relative expression analyses

Nucleated cells of PB and BM samples were separated after centrifugation and red cell lysis, then suspended with Trizol reagent (Invitrogen) for total RNA extraction. Quality assessment and quantification of RNA were conducted with a NanoDrop 2000 spectrophotometer (Thermo Scientific). About 3 μg of total RNA was used for cDNA synthesis in a 20‐µL reaction volume using RevertAid Reverse Transcriptase (Thermo Scientific, USA) on a 7900 HT Fast Real‐Time PCR System (Applied Biosystems). RT‐qPCR was performed in a 20‐µL reaction volume using 1‐µL cDNA with SYBR Green Mix (Thermo Scientific, USA) in an ABI 7500 sequence detection system (Applied Biosystems, USA). GAPDH was used as internal control. Primers for the RT‐qPCR are listed in the Supporting Information. All samples were run in duplicate. Comparative ΔΔCt method was used with one of the healthy donors as the calibrator. hKLF3 relative expression (2^‐ΔΔCt^) was calculated as below:

$$\Delta \text{Ct}\left(\text{hKLF}3\right)=\text{Ct}\left(\text{hKLF}3\right)-\text{Ct}\left(\text{GAPDH}\right)$$

$$\Delta \Delta \text{Ct}\left(\text{hKLF}3\right)=\Delta \text{Ct}\left(\text{hKLF}3\right)-\Delta \text{Ct}\left(\text{calibrator}\right)$$

### 2.4 Statistical analysis

Data were presented as median (range) or mean ± SD. Differences between two independent groups were tested with the Mann‐Whitney *U* test (continuous variables and nonparametric analyses). Receiver operating characteristic (ROC) curve was constructed and area under the curve (AUC) with 95% CI was calculated to assess sensitivity and specificity. We investigated the optimum cutoff value for diagnosis by balancing the maximization of the Youden index (sensitivity + specificity − 1), sensitivity, and specificity. Analyses of the frequencies were performed using the Pearson χ^2^ test for 2 × 2 tables. Statistical analysis of data from paired samples was performed with Wilcoxon's signed‐rank test. Correlation between independent data was tested with Spearman's correlation test. Data analyses and graphic rendering were performed with SPSS 21.0 and GraphPad Prism 6.0 for Mac, respectively. Two‐tailed *P* values <.05 were considered statistically significant.

## 3 RESULTS

### 3.1 hKLF3 expression in PB at diagnosis of AL

**Figure 1:**
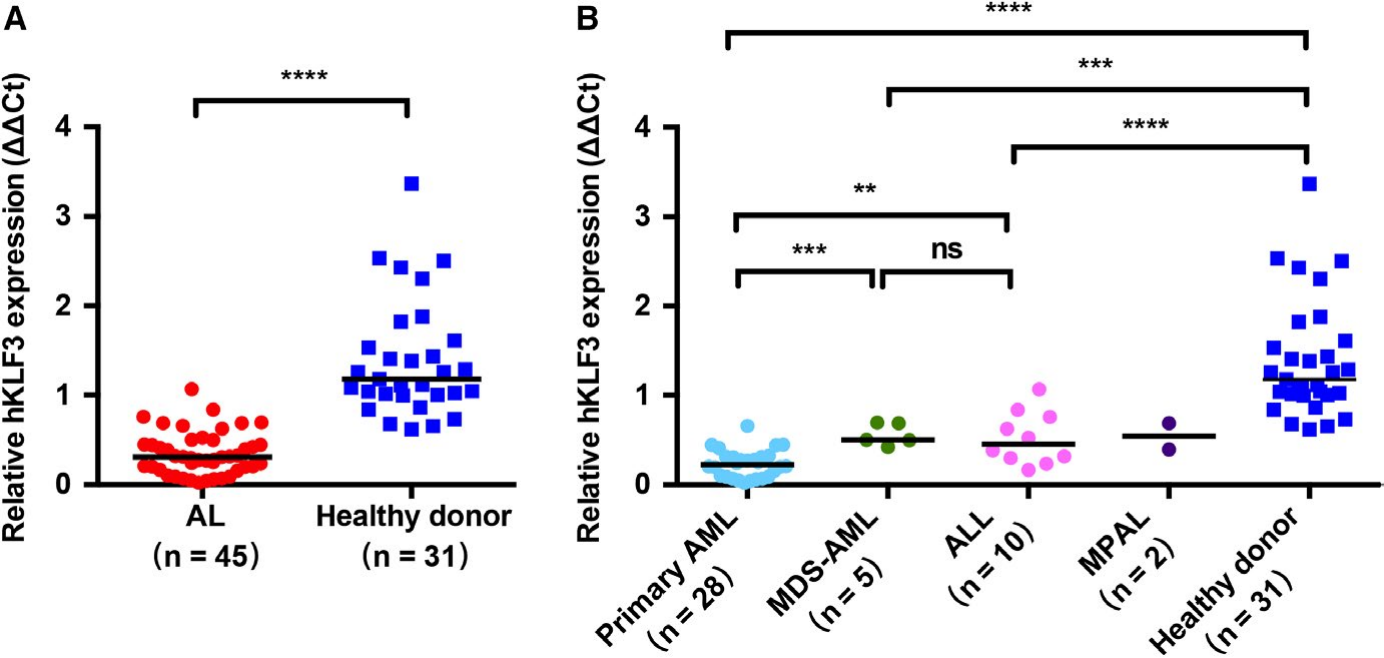
hKLF3 expression in peripheral blood (PB) in different groups. A, Comparison of hKLF3 expression in AL patients and in healthy controls. B, Comparison of hKLF3 expression in healthy controls and AL subgroups, including primary AML, secondary AML (MDS‐AML), and ALL. Abbreviations: MDS‐AML, AML secondary to MDS; MPAL, mixed phenotypic acute leukemia. Horizontal bars indicated the median values of each group. ****P < .0001, ***P < .0005, **P < .005, ns, no significance

In the sequence analyses of hKLF3 coding exons, we observed no gene mutation in BM samples from 14 AML and three ALL patients. The relative expression level of hKLF3 in the AL patients at diagnosis was 0.30 (0.02‐1.07), which was significantly lower than that in the healthy donors [1.18 (0.62‐3.37), *P* < .0001, Figure 1A].

According to the origin of leukemia lineage types, the AL patients were divided into AML, ALL, and MPAL. AML was subsequently divided into primary AML and AML secondary to myelodysplasia syndrome (MDS). In the subsets, primary AML patients showed lower hKLF3 expression level in PB [0.23 (0.02‐0.81)] than that in AML secondary to MDS [0.50 (0.42‐0.69), *P* = .0002] and ALL [0.45 (0.16‐1.07), *P* = .0034)]. We observed almost equivalent hKLF3 expression in patients of AML secondary to MDS and ALL (*P* = .6753) (Figure 1B). Additionally, hKLF3 expression levels in all the above three subgroups were lower than that in healthy donors (*P* < .0001, *P* = .0002, and *P* < .0001, respectively) (Figure 1B).

### 3.2 hKLF3 expression in PB as a potential marker for AL diagnosis

**Figure 2:**
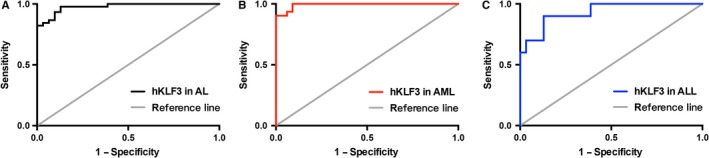
The ROC analyses of hKLF3 expression in PB for diagnosing AL (A), AML (B), and ALL (C)

Given that hKLF3 is expressed widely in normal hematopoietic cells, it is essential to establish threshold to distinguish AL from background amplification. We performed ROC analysis in the AL patients and the healthy donors, which showed that the AUC value for AL was 0.98 (95% CI 0.95‐1.00), and the optimum cutoff value was 0.71 (sensitivity 93.33% and specificity 90.32%) (Figure 2A, Table 2).

**Table 2:** Diagnostic power of hKLF3 expression for AL

	AUC	95% CI	Cutoff value	Youden index	Sensitivity (%)	Specificity (%)	Positive LR	Negative LR
AL vs donors	0.98	0.95‐1.00	0.71	0.84	93.33	90.32	9.64	0.07
AML vs donors	0.99	0.98‐1.00	0.67	0.87	93.94	93.55	14.56	0.06
ALL vs donors	0.93	0.85‐1.00	0.84	0.78	90.00	87.10	6.98	0.11

Abbreviations: AL, acute leukemia; ALL, acute lymphoblastic leukemia; AML, acute myeloid leukemia; AUC, area under the curve; CI, confidence interval; hKLF3, human Krüppel‐like factor 3; LR, likelihood ratio.

We learned that hKLF3 expressed less in AML patients than ALL patients, so a better role of this factor in distinguishing AML patients from healthy controls was anticipated. Compared to the AUC of 0.93 (95% CI 0.85‐1.00) in ALL subgroup (Figure 2C), AML patients displayed a superior AUC of 0.99 (95% CI 0.98‐1.00) (Figure 2B). With 0.67 defined as the optimum cutoff value of the AML subgroup, the corresponding sensitivity and specificity were 93.94% and 93.55%, respectively (Figure 2B, Table 2). These results indicated good performance of hKLF3 expression in PB for the diagnosis of AML.

### 3.3 hKLF3 expression in PB at morphological CR

**Figure 3:**
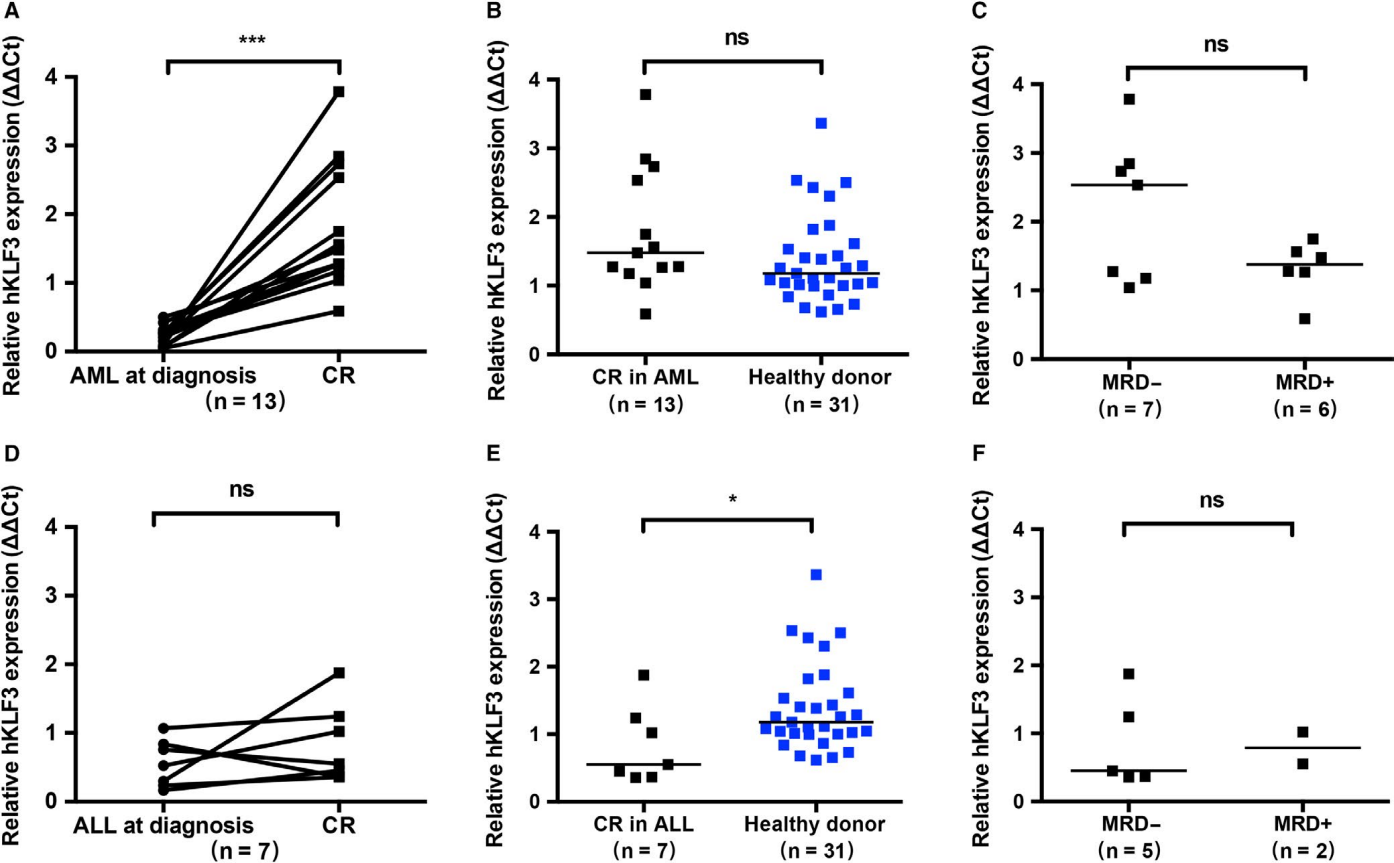
hKLF3 expression status in peripheral blood (PB) of AML and ALL patients at morphological CR. A, Upregulation of hKLF3 expression in AML patients at morphological CR, compared with the level at diagnosis. B, Comparison of hKLF3 expression levels between AML patients at morphological CR and the healthy controls. C, Comparison of hKLF3 expression levels in AML patients between MRD‐negative (MRD‐) subgroup and MRD‐positive (MRD+) subgroup. D, Analysis of hKLF3 expression in matched PB samples of ALL patients at diagnosis and after morphological CR. E, Comparison of hKLF3 expression levels between ALL patients at morphological CR and the healthy controls. F, Comparison of hKLF3 expression levels in ALL patients between MRD‐ subgroup and MRD + subgroup. Horizontal bars indicated the median values of each group. ***P < .0005, *P < .05, ns. no significance

PB samples were collected from 13 AML patients and seven ALL patients who had achieved morphological CR during the follow‐up. Comparison of the samples collected at diagnosis and matched samples collected at morphological CR revealed that the expression of hKLF3 in PB of AML patients increased significantly to 1.48 (0.59‐3.79) after morphological CR (*P* = .0002, Figure 3A). The expression values of hKLF3 in more than 90% of these samples exceeded the threshold value of 0.67 which was defined by PB samples collected from healthy donors, and no significant difference was observed between these patients and the healthy donors (*P* = .0848, Figure 3B).

During chemotherapy, the AML patients obtained deeper remission with cycles of intensive induction and consolidation therapy, and MRD level detected through FCM gradually decreased. The values of hKLF3 expression in MRD‐negative AML patients [2.54 (1.04‐3.79)] appeared to be higher than those in MRD‐positive AML patients [1.38 (0.59‐1.75)] (Figure 3C). However, no statistical significance was observed (*P* = .3566).

On the contrary, for ALL patients, no significant upregulation of hKLF3 expression was observed at morphological CR, compared with hKLF3 level at diagnosis (*P* = .3750, Figure 3D); and hKLF3 level of these patients at morphological CR was still significantly lower than that in the healthy donors (*P* = .0254, Figure 3E). Additionally, no difference was observed between MRD‐negative subgroup and MRD‐positive subgroup (*P* = .8571, Figure 3F).

### 3.4 Comparison of hKLF3 expression in paired PB and BM samples

**Figure 4:**
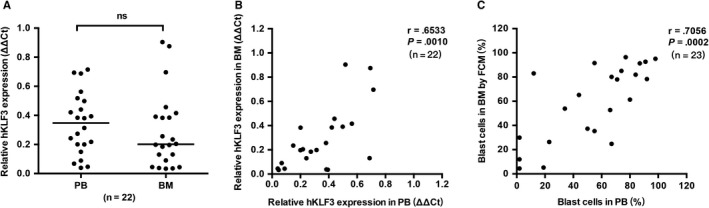
hKLF3 expression in paired peripheral blood (PB) and bone marrow (BM) samples. A, Comparison of hKLF3 expression in paired PB and BM samples. B, Correlation of hKLF3 expression between paired PB and BM samples. C, Correlation of the blast cell percentage in paired PB and BM samples. Horizontal bars indicated the median values of each group. ns. no significance

The expression of hKLF3 in BM samples was obtained in 22 AL patients at the time of diagnosis. The comparison of hKLF3 expression levels in paired pretreatment PB and BM samples showed the tendency that BM samples expressed less hKLF3 transcripts than PB samples [0.20 (0.03‐0.90) vs 0.35(0.04‐0.71)]; however, no significant difference was observed (*P* = .1375, Figure 4A). What's more, hKLF3 expression in the paired samples was observed to be closely correlated (*r* = .6533, *P* = .0010, Figure 4B), which was in accordance with the correlation between percentages of the blast cells in PB and BM samples (*r* = .7056, *P* = .0002, Figure 4C).

### 3.5 Correlation of hKLF3 expression in PB with leukemic load

**Figure 5:**
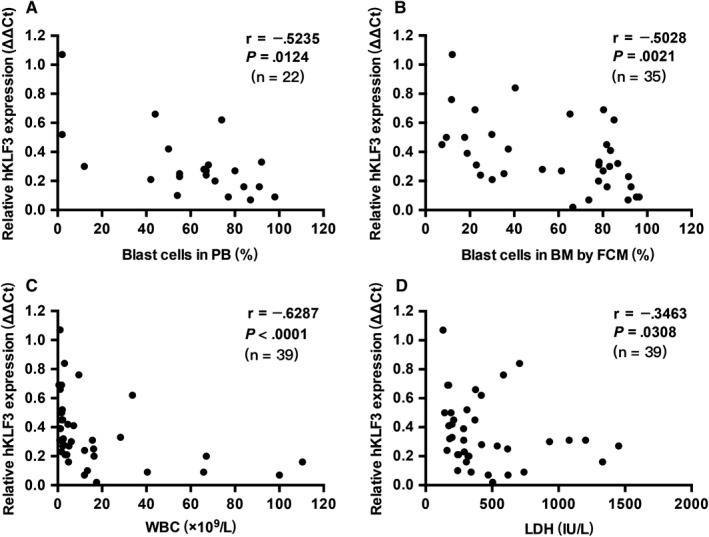
The converse correlation between hKLF3 expression level and markers for leukemic load, including percentages of blast cells in peripheral blood (A), bone marrow (B), peripheral white blood cell count (C), and serum lactate dehydrogenase (D)

To investigate the relationship between the values of hKLF3 expression in PB and the leukemic load, we analyzed the clinical data of the AL patients. We observed that the decreased hKLF3 expression value in PB at diagnosis was negatively correlated with the percentage of blast cells in PB samples (*r* = −.5235, *P* = .0124, Figure 5A) and BM samples (*r* = −.5028, *P* = .0021, Figure 5B). That is, the more leukemia cells, the lower hKLF3 expression. Besides, hKLF3 expression was found to be correlated more closely with peripheral white blood cell (WBC) count (*r* = −.6287, *P* < .0001, Figure 5C), which generally denotes extremely active tumor proliferation and highly malignant tendency. As we all know, serum lactate dehydrogenase (LDH) is generally released from destructed tumor cells and is considered to be a reliable indicator of tumor burden. A mild correlation was also observed between LDH and hKLF3 expression in PB (*r* = −.3463, *P* = .0308, Figure 5D).

## 4 DISCUSSION

In the present study, we have investigated the expression of hKLF3 in AL patients by both Sanger sequencing and RT‐qPCR. No gene mutation was detected in hKLF3 coding exons of AL patients, and hKLF3 expression level in PB samples of AML and ALL patients was significantly lower than that in healthy donors. ROC analyses indicated excellent efficacy of hKLF3 expression in PB samples for the diagnosis of AML. When the AML patients acquired morphological CR, the expression of hKLF3 returned to normal level, and AML patients who obtained deeper CR without MRD had relatively higher hKLF3 level than AML patients with MRD‐positive. Comparison of data from paired PB and BM samples revealed consistent change between the two types of samples. What's more, we found a significant converse correlation between inhibition of hKLF3 expression and increased leukemic burden.

KLF3 is a member of the Krüppel‐like factor (KLF) transcription factor family. The KLFs consist of at least 17 members characterized by the presence of three highly conserved C‐terminal C2H2 zinc fingers that allow for its sequence‐specific binding onto CACCC boxes and GC‐rich motifs in the promoters and control regions of its target genes.^3^ Its N‐terminal region is more variable to interact with diverse co‐regulator proteins. KLF3 was first identified in an erythroid screen for factors related to KLF1 in 1996,^5^ and hKLF3 was firstly cloned in our lab from the cDNA library of fetal liver.^2^ KLF3 recruits the transcriptional corepressor C‐terminal‐binding protein (CtBP) to the promoters of target genes and acts mainly as a repressor of transcription.^6^, ^7^ Currently known biological functions of KLF3 include regulation of adipogenesis and fatty acid metabolism,^8^, ^9^ muscle cell biology,^10^ erythropoiesis,^11^ and B‐cell development.^6^, ^12^, ^13^, ^14^

Highly conserved C‐terminal structure and conservative binding site with CACCC boxes or GC‐rich motifs lead to a certain degree of overlap in the target genes that different KLFs regulate.^3^ Several KLFs have been reported to be involved in malignant diseases,^15^, ^16^, ^17^ which is in accordance with their fundamental role in the regulation of cell growth, proliferation, differentiation, and apoptosis. KLF2 appeared to inhibit cell proliferation by decreasing the expression of proto‐oncogene c‐myc,^18^ and mutations in KLF2 inactivated its ability to suppress NF‐κB activation.^19^ KLF3 was shown to be involved in the formation of B‐cell cancers in vivo, and the KLF3 locus was a common integration site for B‐cell tumorigenesis.^6^, ^20^ Low levels of KLF4 transcripts were detected in AML, T‐cell ALL, and lymphomas,^21^, ^22^, ^23^ suggesting its potential tumor‐suppressive properties.^24^ KLF13 was downregulated in prostate tumor tissues, and overexpression of KLF13 inhibited cell proliferation and induced apoptosis by suppressing the AKT pathway in human prostate cancer cells.^25^ To date, little has been known about the role of KLF3 in leukemia pathogenesis.

Initiation and maintenance of AL are based on the uncontrolled proliferation and arrest of differentiation of the leukemic blasts. We believe that the expression of hKLF3 in PB could help to distinguish the abnormally proliferating blasts from their normal counterparts. Our results indicated that hKLF3 expression was inhibited in AML and ALL, which was in line with the findings in acute promyelocytic leukemia (APL) patients by Magali Humbert et al.^26^ And, ROC analyses indicated excellent efficacy of this factor for AL diagnosis, especially for the diagnosis of AML. Therefore, hKLF3 could serve as a potential auxiliary marker for AL diagnosis. What's more, the expression of hKLF3 was observed to be upregulated to normal range when the AML patients acquired morphological CR, and the hKLF3 level seemed to be higher in patients with deeper CR than in MRD‐positive patients, which was in accordance with the converse correlation between hKLF3 expression values and leukemic burden. Therefore, the factor could also be used to monitor response to chemotherapy and residual disease. Though the sample size of this research is not large enough, the results are significant and instructive for future research.

As an extensively expressed transcription factor, hKLF3 was thought to provide little information on differentiating lineage origin of tumor cells. Wilms tumor gene 1 (WT1), a typical universal marker, is usually used to distinguish neoplastic diseases from benign disorders and monitor MRD dynamically.^1^, ^27^ We believe hKLF3 would be another potential universal marker which can help to distinguish blasts from normally differentiated cells and tissues. In our study, subgroups of AL patients displayed different hKLF3 expression levels, which was possibly the result of different pathogenesis and pathology. But our data only studied a small number of patients with AML and ALL, and we did not detect the expression profiles of hKLF3 in other tumors, so hKLF3 cannot be used for the differential diagnosis among leukemia subgroups or to distinguish AL from other tumors. A larger cohort of patients and controls needs to be studied to validate the findings, and more types of tumors, such as chronic lymphocytic leukemia, chronic myeloid leukemia, and lymphoma involving BM, should be included in future study to further evaluate the role of hKLF3 as a good universal marker.

The specific molecular roles of hKLF3 in leukemia pathogenesis remain to be further investigated. hKLF3 was assumed to exert tumor‐suppressive effects through transcriptional regulation of its target genes, which involved in the cellular proliferation and differentiation, and thus inhibition of its expression would lead to tumorigenesis. We sequenced no gene mutations in coding exons that were responsible to the altered expression of this factor. On the other hand, it is thought that malignant growth is characterized with reprogrammed utilization of glycolysis to fulfil anabolic demands.^28^ Leukemic blasts display high glucose consumption and heavily rely on it,^29^ and impairment of glycolysis strongly inhibits leukemia initiation and maintenance.^30^ In normal hematopoietic stem cells, the switch from aerobic glycolysis to oxidative phosphorylation is necessary to abolish the self‐renewal capacity and maintain lineage differentiation process.^31^, ^32^ KLF3 has been identified to interact with several genes encoding fat metabolic enzymes in modulating fatty acid β‐oxidation in mitochondria,^9^ and therefore we assume that hKLF3 is downregulated in leukemic blasts to maintain optimal glycolysis‐based energy metabolism mode that helps the leukemic blasts to grow and divide faster and more efficiently than their normal counterparts, thus becoming the dominant group in BM. Yet we cannot deny the possibility that the factor did not participate directly in the regulation of leukemic pathogenesis, and the altered hKLF3 expression was merely an accompanying product during the process of oncogenesis. In whichever condition, hKLF3 showed its potential role as a promising biomarker for leukemic blasts. In future study, we are supposed to conduct hKLF3 detection in each cell subgroup after separating tumor and normal cells in samples from leukemia patients. Exploration of underlying mechanism of the altered expression of hKLF3 would make the factor a potential therapeutic target.

In our study, the expression of hKLF3 in BM samples seemed to be lower than that in PB samples, but the difference failed to reach statistical significance. As we all know, hematopoietic cells in BM generally stay in early stages and keep active proliferation, therefore, the background amplification of hKLF3 in BM is speculated to be lower than that in the leukocytes of PB, which have differentiated into mature and functional stage. When normal hematopoiesis is inhibited by leukemic blasts infiltration, hKLF3 expression in PB decreases significantly, while hKLF3 expression in BM would not be deregulated that much due to the preexisting lower baseline. More importantly, hKLF3 expression levels in the paired PB and BM samples were found to be closely correlated and exhibited the consistent changing pattern. While BM specimens are currently the main objects for molecular detection methods, our data suggested that the use of PB samples might be a good alternative in detection of hKLF3 expression for assessment of treatment response and MRD monitoring. PB sampling is minimally invasive, and thus leads to better patient compliance meanwhile reducing the consumption of medical resources.

In conclusion, we identified the potential role of hKLF3 expression in PB as a diagnostic and monitoring marker for AL, particularly for AML patients who were in critical shortage of specific molecular targets. In future study, the role of hKLF3 expression in MRD monitoring is still to be evaluated.

## CONFLICT OF INTEREST

The authors declare no competing interest.

## AUTHOR CONTRIBUTIONS

All authors contributed to the study design and conduction. Miao Yan, Mangju Wang, Ping Zhu, Hanyun Ren, and Fuchu He designed the research. Miao Yan, Huihui Liu, Junhui Xu, Qian Wang, Weilin Xu, Xinan Cen, Wensheng Wang, Zhixiang Qiu, Jinping Ou, and Yujun Dong collected the samples and performed the Sanger sequencing and RT‐qPCR. Miao Yan, Hanyun Ren, and Mangju Wang analyzed the data and wrote the paper. All authors reviewed and revised the manuscript.

## Data Availability

The data that support the findings of this study are available from the corresponding author upon reasonable request.

## References

[1] Cilloni D , Renneville A , Hermitte F , et al. Real‐time quantitative polymerase chain reaction detection of minimal residual disease by standardized WT1 assay to enhance risk stratification in acute myeloid leukemia: a European LeukemiaNet study. J Clin Oncol. 2009;27(31):5195‐5201. 19752335 10.1200/JCO.2009.22.4865

[2] Wang MJ , Qu XH , Wang LS , et al. cDNA cloning, subcellular localization and tissue expression of a new human Krüppel‐like transcription factor: human basic Krüppel‐like factor (hBKLF). Acta Genetica Sinica. 2003;30(1):1. 12812068

[3] Dang DT , Pevsner J , Yang VW . The biology of the mammalian Krüppel‐like family of transcription factors. Int J Biochem Cell Biol. 2000;32(11):1103‐1121. 11137451 10.1016/s1357-2725(00)00059-5 PMC2754176

[4] Wang M , Ma XY , Shi YJ , et al. Effect of the new human transcription factor hBKLF on the proliferation, differentiation of K562 cell line and hemoglobin synthesis. Journal of Experimental Hematology. 2006;14(6):1083‐1088. 17204169

[5] Crossley M , Whitelaw E , Perkins A , et al. Isolation and characterization of the cDNA encoding BKLF/TEF‐2, a major CACCC‐box‐binding protein in erythroid cells and selected other cells. Mol Cell Biol. 1996;16(4):1695‐1705. 8657145 10.1128/mcb.16.4.1695 PMC231156

[6] Life I . The mammalian zinc finger transcription factor Krüppel‐like factor 3 (KLF3/BKLF). IUBMB Life. 2015;63(2):86‐93. 10.1002/iub.422 21360637

[7] Dewi V , Kwok A , Lee S , et al. Phosphorylation of Kruppel‐like factor 3 (KLF3/BKLF) and C‐terminal binding protein 2 (CtBP2) by homeodomain interacting protein Kinase 2 (HIPK2) modulates KLF3 DNA binding and activity. J Biol Chem. 2015;290(13):8591‐8605. 25659434 10.1074/jbc.M115.638338 PMC4375508

[8] Nancy S , Jack BHA , Eaton SA , et al. Targeted disruption of the basic Krüppel‐like factor gene (Klf3) reveals a role in adipogenesis. Mol Cell Biol. 2008;28(12):3967‐3978. 18391014 10.1128/MCB.01942-07 PMC2423134

[9] Zhang J , Bakheet R , Parhar R , et al. Regulation of fat storage and reproduction by Kruppel‐like transcription factor KLF3 and fat‐associated genes in Caenorhabditis elegans. J Mol Biol. 2011;411(3):537‐553. 21704635 10.1016/j.jmb.2011.06.011 PMC4371853

[10] Himeda CL , Ranish JA , Pearson RCM , et al. KLF3 regulates muscle‐specific gene expression and synergizes with serum response factor on KLF binding sites. Mol Cell Biol. 2010;30(14):3430‐3443. 20404088 10.1128/MCB.00302-10 PMC2897560

[11] Funnell APW , Norton LJ , Ka Sin M , et al. The CACCC‐binding protein KLF3/BKLF represses a subset of KLF1/EKLF target genes and is required for proper erythroid maturation in vivo. Mol Cell Biol. 2012;32(16):3281‐3292. 22711990 10.1128/MCB.00173-12 PMC3434552

[12] Thi Thanh V , Dominique G , Vivian T , et al. Impaired B cell development in the absence of Krüppel‐like factor 3. Journal of Immunology. 2011;187(10):5032‐5042. 10.4049/jimmunol.1101450 22003205

[13] Hart GT , Hogquist KA , Jameson SC . Krüppel‐like Factors in Lymphocyte Biology. Journal of Immunology. 2012;188(2):521‐526. 10.4049/jimmunol.1101530 PMC3257580 22223851

[14] Gleb T , Thi Thanh V , Friederike F , et al. Programming of marginal zone B‐cell fate by basic Kruppel‐like factor (BKLF/KLF3). Blood. 2011;117(14):3780‐3792. 21297003 10.1182/blood-2010-09-308742

[15] Zhang Y , Hao J , Zheng Y , et al. Role of Krüppel‐like factors in cancer stem cells. J Physiol Biochem. 2015;71(1):155‐164. 25616500 10.1007/s13105-015-0381-4

[16] Ridha L , Ken ODB , Filip L , et al. Krüppel‐like factors in cancer progression: three fingers on the steering wheel. Oncotarget. 2013;5(1):29‐48. 10.18632/oncotarget.1456 PMC3960187 24429391

[17] Memon A , Lee W . KLF10 as a tumor suppressor gene and Its TGF‐β signaling. Cancers. 2018;10(6):161. 10.3390/cancers10060161 PMC6025274 29799499

[18] Buckley AF , Kuo CT , Leiden JM . Transcription factor LKLF is sufficient to program T cell quiescence via a c‐Myc‐dependent pathway. Nat Immunol. 2001;2(8):698‐704. 11477405 10.1038/90633

[19] Clipson A , Wang M , De LL , et al. KLF2 mutation is the most frequent somatic change in splenic marginal zone lymphoma and identifies a subset with distinct genotype. Leukemia. 2015;29(5):1177‐1185. 25428260 10.1038/leu.2014.330

[20] Keiko A , Takeshi S , Stephens RM , et al. RTCGD: retroviral tagged cancer gene database. Nucleic Acids Res. 2004;32(90001):D523‐D527. 14681473 10.1093/nar/gkh013 PMC308748

[21] Jun‐Ichirou Y , Yuko T , Kisato N , et al. Identification of aberrantly methylated genes in association with adult T‐cell leukemia. Can Res. 2004;64(17):6002‐6009. 10.1158/0008-5472.CAN-04-1422 15342380

[22] Guan H , Xie LF , Flossbach L , et al. KLF4 is a tumor suppressor in B‐cell non‐Hodgkin lymphoma and in classic Hodgkin lymphoma. Blood. 2010;116(9):1469. 20519630 10.1182/blood-2009-12-256446

[23] Katrin F , Lars B , Christine R , et al. CDX2‐driven leukemogenesis involves KLF4 repression and deregulated PPARγ signaling. J Clin Invest. 2013;123(1):299‐314. 23202735 10.1172/JCI64745 PMC3533294

[24] Park CS , Lewis A , Chen T , et al. Concise review: Regulation of self‐renewal in normal and malignant hematopoietic stem cells by Krüppel‐like factor 4. Stem Cells Transl Med. 2019;8(6):568‐574. 30790473 10.1002/sctm.18-0249 PMC6525558

[25] Wang Q , Peng R , Wang B , et al. Transcription factor KLF13 inhibits AKT activation and suppresses the growth of prostate carcinoma cells. Cancer Biomark. 2018;22(3):1‐9. 29843216 10.3233/CBM-181196 PMC6218114

[26] Humbert M , Halter V , Shan D , et al. Deregulated expression of Kruppel‐like factors in acute myeloid leukemia. Leuk Res. 2011;35(7):909‐913. 21470678 10.1016/j.leukres.2011.03.010

[27] Nomdedeu JF , Hoyos M , Carricondo M , et al. Bone marrow WT1 levels at diagnosis, post‐induction and post‐intensification in adult de novo AML. Leukemia. 2013;27(11):2157‐2164. 23584566 10.1038/leu.2013.111

[28] Hay N . Reprogramming glucose metabolism in cancer: can it be exploited for cancer therapy? Nat Rev Cancer. 2016;16(10):635. 27634447 10.1038/nrc.2016.77 PMC5516800

[29] Kreitz J , Schonfeld C , Seibert M , et al. Metabolic plasticity of acute myeloid leukemia. Cells. 2019;8(8):805. 10.3390/cells8080805 PMC6721808 31370337

[30] Wang Y , Israelsen WJ , Lee D , et al. Cell‐state‐specific metabolic dependency in hematopoiesis and leukemogenesis. Cell. 2014;158(6):1309‐1323. 25215489 10.1016/j.cell.2014.07.048 PMC4197056

[31] Yu WM , Liu X , Shen J , et al. Metabolic regulation by the mitochondrial phosphatase PTPMT1 is required for hematopoietic stem cell differentiation. Cell Stem Cell. 2013;12(1):62‐74. 23290137 10.1016/j.stem.2012.11.022 PMC3632072

[32] Takubo K , Nagamatsu G , Kobayashi CI , et al. Regulation of glycolysis by Pdk functions as a metabolic checkpoint for cell cycle quiescence in hematopoietic stem cells. Cell Stem Cell. 2013;12(1):49‐61. 23290136 10.1016/j.stem.2012.10.011 PMC6592822

