# Supplementary material for: Expression of human Krüppel‐like factor 3 in peripheral blood as a promising biomarker for acute leukemia
Source: Cancer Med. 2020 Feb 26;9(8):2803–11. doi: 10.1002/cam4.2911 (PMC7163096; doi:10.1002/cam4.2911)
Supplement: Supplementary file 1 [file CAM4-9-2803-s001.pdf]

Supplementary materials

Primers for amplification

Primers	Sequence
hKLF3_Exon2-F	5′-CTCACTGCAACCTCCGCCTCCT-3′
hKLF3_Exon2-R	5′-TCCCGCCTCGGTCTCCCAAA-3′
hKLF3_Exon3-F	5′-TTGGCTTGACTGGTTCTTGT-3′
hKLF3_Exon3-R	5′-ACAGACAGCCTGATTCTTGG-3′
hKLF3_Exon4-F	5′-TTTCAGGGCTGGTGTCTTA-3′
hKLF3_Exon4-R	5′-ATTCAGGTGCTTGGATGCT-3′
hKLF3_Exon5-F	5′-CACTTGGTGCCTGCCTCTA-3′
hKLF3_Exon5-R	5′-CCTTTCTCCCTCCTTTGCT-3′
hKLF3_Exon6-F	5′-TTTGGTAGTCCCTCAGAATG-3′
hKLF3_Exon6-R	5′-TGTGAAGAATGACGGAAGAG-3′

Primers for sequencing

Primers	Sequence
hKLF3_Exon2-F1	5′-ATTATAGACGAGTGCCATCACG-3′
hKLF3_Exon3-R1	5′-CCCCATGCTTCCTCACCCAC-3′
hKLF3_Exon4-R1	5′-CACAGATCACAAGCCGTTCC-3′
hKLF3_Exon5-R1	5′-CAAAGGCAAATCATTAGCAATA-3′
hKLF3_Exon6-R1	5′-GTCTGACCCGTGGTAAGGAG-3′

Primers for RT-qPCR

Primers	Sequence
hKLF3-F	5′-TGTCTCAGTGTCATACCCATCT-3′
hKLF3-R	5′-CCTTCTGGGGTCTGAAAGAACTT-3′
GAPDH-F	5′-CATCCATGACAACTTTGGTATCGT-3′
GAPDH-R	5′-CCATCACGCCACAGTTTCC-3′
